# Temporal evolution of the LI-RADS radiation treatment response assessment on multiphase CT/MRI in patients undergoing selective internal radiation therapy for hepatocellular carcinoma

**DOI:** 10.1007/s00330-025-11659-1

**Published:** 2025-05-17

**Authors:** Hong Wei, Hanyu Jiang, Jeongin Yoo, Jae Hyun Kim, Hyo-Jin Kang, Yuanan Wu, Rongbo Liu, Hyo-Cheol Kim, Jeong Min Lee

**Affiliations:** 1 Department of Radiology, Seoul National University Hospital, Seoul, Republic of Korea; 2 Department of Radiology, Functional and Molecular Imaging Key Laboratory of Sichuan Province, West China Hospital, Sichuan University, Chengdu, China; 3 Department of Radiology, Seoul National University College of Medicine, Seoul, Republic of Korea; 4 Big Data Research Center, University of Electronic Science and Technology of China, Chengdu, China

**Keywords:** Carcinoma (hepatocellular), Selective internal radiation therapy, Liver imaging reporting and data system treatment response, Computed tomography, Magnetic resonance imaging

## Abstract

**Objectives:**

To assess the temporal evolution and interobserver agreement of the early categories per the liver imaging reporting and data system (LI-RADS) radiation treatment response assessment (TRA) algorithm in patients receiving selective internal radiation therapy (SIRT) with Yttrium-90 for hepatocellular carcinoma (HCC).

**Materials and methods:**

This single-center retrospective study included consecutive patients with treatment-naïve HCC who underwent serial contrast-enhanced CT/MRI before and after SIRT. Three masked radiologists independently evaluated response at 3–6 months. Another senior radiologist assessed response at 9, 12, 15, 18, 21, 24, and > 24 months after comprehensive review of available clinical-radiological information.

**Results:**

65 patients (mean age, 66.7 ± 11.2 years; 48 men) were included. At 3–6 months after SIRT, 47.7% (31/65) of lesions were assigned to the nonprogressing category, and the remaining 52.3% (34/65) to the nonviable category. Among early nonprogressing lesions, 64.5% (20/31) regressed to the nonviable category, 25.8% (8/31) remained nonprogressing, and 9.7% (3/31) evolved into the viable category at ≥ 12 months. The nonprogressing category decreased in number over time, with 61.3% (19/31) conversion to the nonviable category at 9 months. Among the early nonviable lesions, 91.2% (31/34) remained nonviable at ≥ 12 months, and 8.8% (3/34) evolved into the viable category. Agreement for the 3–6 months LR-TR category assignment was moderate (kappa = 0.46) with CT but almost perfect (kappa = 0.85) with MRI.

**Conclusions:**

SIRT induced a delayed and sustained response in the majority of HCC patients after ≥ 12 months. MRI demonstrated superior agreement over CT in assessing response at 3–6 months.

**Key Points:**

***Question*** *Tumor response to SIRT can change; there is limited evidence on the evolution of the imaging appearance of HCC following SIRT*.

***Findings*** *Sixty-four and five-tenths of early nonprogressing lesions regressed to nonviable, and 91.2% of early nonviable lesions remained free of viability. LR-TR category assignment agreement was moderate with CT but almost perfect with MRI*.

***Clinical relevance*** *SIRT induced a delayed and sustained response in HCC, underscoring the necessity of dynamic evaluation of long-term changes in treated lesions. MRI with subtraction imaging may be preferred over CT for long-term monitoring, which may help prevent premature retreatment decisions*.

**Graphical Abstract:**

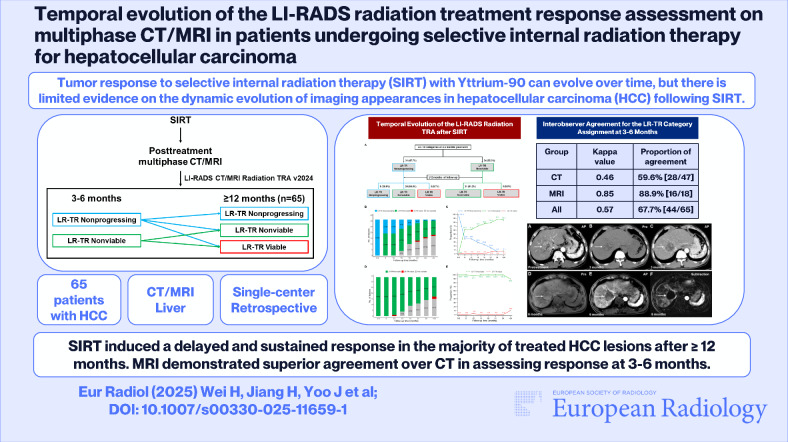

## Introduction

Selective internal radiation therapy (SIRT) with Yttrium-90 (Y90) is an emerging locoregional therapy (LRT) for hepatocellular carcinoma (HCC) [1]. It has demonstrated significant clinical benefits and satisfactory safety profiles across various clinical contexts, e.g., radiation segmentectomy for very early to early-stage tumors, bridging/downstaging therapy before liver transplantation, and as an alternative to transarterial chemoembolization (TACE) for intermediate-stage HCC [2–6]. With its growing role in clinical practice, accurate assessment of treatment response (TR) is essential for guiding management decisions.

The liver imaging reporting and data system (LI-RADS) treatment response assessment (TRA) algorithm provides standardized imaging criteria for evaluating response to LRT in HCC [7]. Unlike other LRTs (e.g., ablation and TACE), radiation-based LRTs, including SIRT, rarely induce early complete necrosis. Instead, tumor response evolves gradually over months due to DNA damage, free radical formation, and activation of proinflammatory and reparative pathways [8, 9]. Considering the unique response pattern, the recently updated 2024 version of LI-RADS introduces a new and separate algorithm for CT/MRI TRA specifically tailored for radiation-based LRTs [9].

To date, evidence remains limited regarding the temporal evolution of HCC TR following SIRT as per the 2024 version of the LI-RADS Radiation TRA. A noteworthy addition in this update is the LR-TR nonprogressing category, which identifies treated lesions with mass-like enhancement that remain stable or decrease in size over time. Given the gradual nature of TR, dynamic evaluation of imaging findings post-SIRT is imperative for optimizing management strategies, such as identifying treatment failure promptly and accurately. Furthermore, interobserver agreement in categorizing TR and evaluating individual imaging features has yet to be thoroughly investigated.

Therefore, this study aimed to assess the temporal evolution of the early (3–6 months) LI-RADS Radiation TRA version 2024 categories and interobserver agreement on multiphase CT/MRI in patients receiving SIRT for HCC.

## Materials and methods

This single-center retrospective study was approved by the Institutional Review Board of Seoul National University Hospital (No. 2301-168-1402), with a waiver for informed consent.

### Study population

Consecutive patients who underwent SIRT for HCC between March 2012 and September 2021 were retrospectively identified if they fulfilled the following inclusion criteria: (a) age ≥ 18 years; (b) HCC diagnosis based on pathologic examinations or noninvasive imaging criteria per the LI-RADS version 2018 diagnostic algorithm [7]; (c) Barcelona clinic liver cancer (BCLC) stage A, B, or C without extrahepatic metastases; (d) no prior HCC treatment; (e) multiphase contrast-enhanced CT/MRI examinations performed within 2 months before SIRT; (f) at least 12 months of imaging follow-up; and (g) no retreatment within 12 months after SIRT. Exclusion criteria included (a) history of malignancies other than HCC; and (b) infiltrative HCC with ill-defined borders (due to the difficulty in reliable size measurement).

Clinical information (e.g., age, sex, and etiology of liver disease), laboratory indexes (e.g., serum α-fetoprotein [AFP] and protein induced by vitamin K absence-II [PIVKA-II]) available within 1 month before SIRT, and pretreatment tumor characteristics (i.e., size, number, liver lobe involvement, and the extent of portal vein tumor thrombosis [10]) were collected from electronic medical records and Picture Archiving and Communication System.

### SIRT procedure

Candidates for SIRT were determined through multidisciplinary tumor board discussions after considering tumor burden and extent (e.g., ≤ 70% of liver volume, single HCC ≤ 8 cm, and absence of main portal vein tumor thrombosis), liver function (i.e., Child–Pugh A or B7), performance status (i.e., Eastern Cooperative Oncology Group score 0 or 1), and patient preferences [1, 11–13]. SIRT was generally considered for patients with HCC not amenable to surgery or ablation, with a life expectancy of > 3 months [2]. The procedure and dosage methodology have been previously detailed [14–17]. In brief, all patients underwent planning angiography with C-arm cone-beam CT and technetium 99m macroaggregated albumin mapping (Tc-99m MAA) (planar scintigraphy and single-photon emission computerized tomography-CT) before SIRT. The tracer (Tc-99m MAA) was injected into the lobar artery feeding the target tumors or the proper hepatic artery for bilobar disease. SIRT was conducted within 2 weeks after the MAA scan in a more selective manner (i.e., segmental artery or distal branch in most cases) using Y90 glass (Therasphere; Boston Scientific) or resin (SIR-Spheres; SIRTEX) microspheres with personalized dosimetry based on the medical internal radiation dose dosimetry model. The selection between glass and resin microspheres was decided at the operators’ discretion. At least 30% of the functional liver volume was spared from radiation, and the maximum lung dose per treatment was limited to 30 Gy.

Details of the SIRT procedure were collected from electronic medical records.

### Imaging technique

For patients with multiple CT or MRI scans prior to SIRT, the imaging study closest to the SIRT date was used. Both CT and MRI examinations were performed in accordance with the minimum technique specifications outlined in the LI-RADS guidelines [18]. Protocols and scanning parameters for liver CT and MRI are described in Supplementary Material 1 and Table S1.

### Imaging analysis

Three abdominal radiologists (H.W., H.J., and R.L.) with 6 years, 8 years, and 20 years of abdominal MRI experience independently evaluated each patient’s CT or MRI studies performed at 3–6 months after SIRT using the LI-RADS CT/MRI Radiation TRA version 2024 [9]. Baseline CT/MRI before SIRT and early follow-up multiphase CT/MRI at 3–6 months after SIRT were provided. Readers were blinded to clinical information and subsequent follow-up imaging studies (> 6 months).

The following imaging features were evaluated as per LI-RADS Radiation TRA version 2024: (a) sizes of whole and viable lesions (cm); (b) presence or absence of individual TR features, including masslike enhancement, complete lesion disappearance, no lesional enhancement, smooth perilesional enhancement, and parenchymal perfusional changes; (c) presence or absence of ancillary features favoring viability (applicable to MRI), including diffusion restriction and/or mild-moderate T2 hyperintensity, which was new or increased over time after treatment in an area of stable or decreased masslike enhancement; and (d) TR category, including LR-TR nonevaluable, LR-TR nonviable, LR-TR nonprogressing, and LR-TR viable. Definitions of these features and categories can be found at the website of LI-RADS CT/MRI Radiation TRA version 2024 Core [9].

The size of viable lesions was measured to assess changes in mass-like enhancement between baseline and follow-up CT/MRI images at 3–6 months after SIRT. When a lesion displayed high signal intensity on precontrast T1-weighted images, mass-like enhancement was evaluated on dynamic phase subtraction images. Tiebreaking rules were applied when there was uncertainty between two TRA categories, choosing the one indicating lower certainty [9]. For patients with multiple HCCs, the largest tumor was assessed. Discrepancies were resolved with the majority interpretation for binary features and by discussion for ordinal and categorical features.

### Reference standard

The reference standard was established by a senior radiologist (J.M.L.) who had over 20 years of liver MRI experience, according to LI-RADS CT/MRI radiation TRA version 2024 [9]. The reader had access to all available pre- and post-treatment imaging studies, routine radiological reports, and clinical information, including tumor markers such as AFP and PIVKA-II. Follow-up LR-TR categories were assigned at 9, 12, 15, 18, 21, 24, and > 24 months on multiphase CT/MRI. Patients were followed until unequivocal disease progression, retreatment (for nonprogressing residual tumors), or January 22, 2024, whichever occurred first.

### Statistical analysis

Continuous data were summarized as means ± standard deviations or medians with interquartile ranges (IQRs), as appropriate. Categorical data were reported as frequencies and percentages. Sensitivity analyses were performed for patients with ≥ 24 months of imaging follow-up. Interobserver agreement among the three readers based on the early follow-up images was assessed by the Fleiss’ kappa value for binary variables, the weighted Fleiss kappa for categorical variables or against the first kappa paradox [19, 20] for highly skewed binary variables (i.e., those with proportion > 90.0% or < 10.0%), and the intraclass correlation coefficient (ICC) for continuous variables. A kappa or ICC of < 0 indicated poor; 0.01–0.20, slight; 0.21–0.40, fair; 0.41–0.60, moderate; 0.61–0.80, substantial; and > 0.80, almost perfect agreement [21].

Statistical analyses were performed using the R software (version 4.3.1; The R Foundation for Statistical Computing) and IBM SPSS software (version 26.0; SPSS Inc.). Two-tailed *p*-values ≤ 0.05 were considered statistically significant.

## Results

### Patient characteristics

**Fig. 1:**
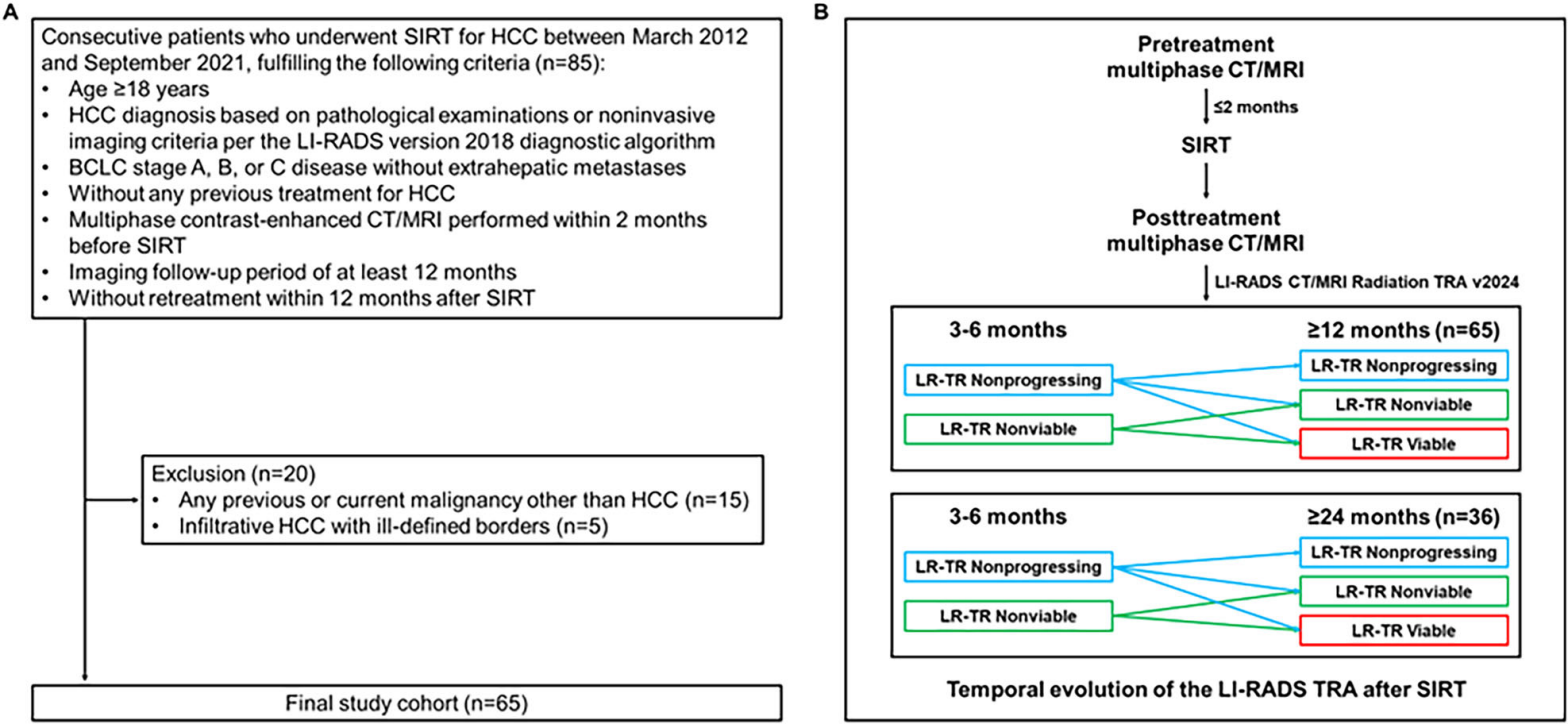
**A** Flowchart of patient enrollment. **B** Study schema. BCLC, Barcelona Clinic Liver Cancer; CT, computed tomography; HCC, hepatocellular carcinoma; LI-RADS/LR, liver imaging reporting and data system; MRI, magnetic resonance imaging; SIRT, selective internal radiation therapy; TR, treatment response; TRA, treatment response algorithm

Among 85 patients treated with SIRT for HCC, 20 were excluded from the study population (Fig. 1).

In total, 65 patients (mean age, 66.7 ± 11.2 years; 48 [73.8%] men) were finally included in the study. Patient characteristics are summarized in Table 1. Chronic hepatitis B was the most common (40 [61.5%]) etiology of HCC, 46 (70.8%) patients had cirrhosis, and 50 (76.9%) patients had Child-Pugh class A liver function. A total of 34 (52.3%) patients were classified as BCLC stage A, 18 (27.7%) as stage B, and 13 (20.0%) as stage C. There were 47 (72.3%), 13 (20.0%), and 5 (7.7%) patients with solitary, 2 or 3, and ≥ 4 HCCs, respectively, with a median largest tumor size of 4.7 cm (IQR, 3.7–6.1 cm). Median interval between SIRT and early follow-up CT/MRI was 3.7 months (IQR, 3.3–4.0 months), and median follow-up period after SIRT was 26.5 months (IQR, 16.4–40.0 months).

**Table 1:** Patients characteristics

Characteristic	Patient (*n* = 65)
Age (y)^*^	66.7 ± 11.2
Sex	
Women	17 (26.2)
Men	48 (73.8)
ECOG performance status	
0	63 (96.9)
1	2 (3.1)
Etiology of liver disease	
HBV	40 (61.5)
HCV	7 (10.8)
Alcohol	4 (6.2)
HBV and HCV coinfection	2 (3.1)
HBV and alcohol	5 (7.7)
HCV and alcohol	1 (1.5)
Unknown	6 (9.2)
Cirrhosis	
Absent	19 (29.2)
Present	46 (70.8)
Child-Pugh class and score	
A5	48 (73.8)
A6	2 (3.1)
B7	1 (1.5)
Missing	14 (21.5)
BCLC stage	
A	34 (52.3)
B	18 (27.7)
C	13 (20.0)
Laboratory index	
Alanine transaminase (IU/L)^†^	26.0 (19.5–36.5)
Aspartate transaminase (IU/L)^†^	31.0 (24.0–43.0)
Total bilirubin (mg/dL)^†^	0.5 (0.4–0.6)
Albumin (g/dL)^*^	4.1 ± 0.4
Platelet (× 10^3^/uL)^†^	157.0 (112.0–194.5)
Prothrombin time (s)^*^	96.7 ± 13.6
International normalized ratio^*^	1.0 ± 0.1
AFP (ng/mL)^†^	14.2 (4.0–187.5)
PIVKA-II (mAU/mL)^†^	308.0 (62.5–1393.5)
Missing	4 (6.2)
Pretreatment tumor characteristics	
Largest tumor size (cm)^†^	4.7 (3.7–6.1)
Number of tumors	
Solitary	47 (72.3)
2 or 3 tumors	13 (20.0)
≥ 4 tumors	5 (7.7)
Liver lobe involvement	
Unilobar	54 (83.1)
Bilobar	11 (16.9)
PVTT	
Vp0	52 (80.0)
Vp1	4 (6.2)
Vp2	4 (6.2)
Vp3	3 (4.6)
Vp4	2 (3.1)
SIRT procedure characteristics	
Mean target tissue dose (Gy)^†^	340.0 (228.0–421.0)
Total administered radiation activity (GBq)^†^	2.9 (1.6–4.5)
Total liver volume (mL)^†^	1195.0 (1000.0–1365.0)
Missing	3 (4.6)
Target liver volume (mL)^†^	475.0 (253.8–700.0)
Missing	3 (4.6)
Tumor volume (mL)^†^	60.0 (30.0–110.0)
Missing	2 (3.1)
Interval between pretreatment CT/MRI and SIRT (days)^†^	15 (11–23)
Interval between SIRT and 3–6 months follow-up CT/MRI (months)^†^	3.7 (3.3–4.0)
Duration of follow-up (months)^†^	26.5 (16.4–40.0)

Unless otherwise indicated, data are numerators, and data in parentheses are percentages

Vp0, absence of invasion of (or tumor thrombus in) the portal vein; Vp1, invasion of (or tumor thrombus in) distal to the second order branches of the portal vein, but not of the second order branches; Vp2, invasion of (or tumor thrombus in) second order branches of the portal vein; Vp3, invasion of (or tumor thrombus in) first order branches of the portal vein; and Vp4, invasion of (or tumor thrombus in) the main trunk of the portal vein and/or contra-lateral portal vein branch to the primarily involved lobe

*AFP* α-fetoprotine, *BCLC* Barcelona Clinic Liver Cancer, *CT* computed tomography, *ECOG* Eastern Cooperative Oncology Group, *HBV* hepatitis B virus, *HCV* hepatitis C virus, *MRI* magnetic resonance imaging, *PIVKA-II* protein induced by vitamin K absence-II, *PVTT* portal vein tumor thrombosis, *SIRT* selective internal radiation therapy

^*^ Data are means ± standard deviations

^†^ Data are medians; data in parentheses are interquartile ranges

### Imaging characteristics on 3–6 months follow-up multiphase CT/MRI

LR-TR categories and features on the 3–6 months follow-up multiphase CT/MRI are summarized in Table 2.

**Table 2:** Imaging characteristics on 3–6 months follow-up CT/MRI

Characteristic	Consensus	Reader 1	Reader 2	Reader 3
LR-TR category				
Viable	0 (0.0)	0 (0.0)	0 (0.0)	0 (0.0)
Nonprogressing	31 (47.7)	29 (44.6)	30 (46.2)	35 (53.8)
Nonviable	34 (52.3)	36 (55.4)	35 (53.8)	30 (46.2)
Masslike enhancement				
Absent	34 (52.3)	36 (55.4)	35 (53.8)	30 (46.2)
Stable	0 (0.0)	0 (0.0)	0 (0.0)	0 (0.0)
Decrease in size	31 (47.7)	29 (44.6)	30 (46.2)	35 (53.8)
Increased in size	0 (0.0)	0 (0.0)	0 (0.0)	0 (0.0)
New	0 (0.0)	0 (0.0)	0 (0.0)	0 (0.0)
Complete lesion disappearance				
Absent	64 (98.5)	64 (98.5)	64 (98.5)	65 (100.0)
Present	1 (1.5)	1 (1.5)	1 (1.5)	0 (0.0)
No lesional enhancement				
Absent	56 (86.2)	52 (80.0)	55 (84.6)	53 (81.5)
Present	9 (13.8)	13 (20.0)	10 (15.4)	12 (18.5)
Smooth perilesional enhancement				
Absent	44 (67.7)	43 (66.2)	41 (63.1)	47 (72.3)
Present	21 (32.3)	22 (33.8)	24 (36.9)	18 (27.7)
Parenchymal perfusional changes				
Absent	5 (7.7)	8 (12.3)	8 (12.3)	4 (6.2)
Present	60 (92.3)	57 (87.7)	57 (87.7)	61 (93.8)
Whole lesion size (cm)^†^	4.6 (3.5–5.5)	4.0 (3.0–5.5)	4.7 (3.7–5.9)	4.4 (3.6–6.0)
Viable lesion size (cm)^†^	0.0 (0.0–1.2)	0.0 (0.0–1.4)	0.0 (0.0–1.8)	0.7 (0.0–1.5)

Unless otherwise indicated, data are numerators, and data in parentheses are percentages

*CT* computed tomography, *LR* liver imaging reporting and data system, *MRI* magnetic resonance imaging, *TR* treatment response

^†^ Data are medians; data in parentheses are interquartile ranges

Briefly, based on the consensus interpretations, 47.7% (31/65) of treated lesions were assigned to the LR-TR nonprogressing category, and the remaining 52.3% (34/65) to the LR-TR nonviable category. Since all three readers recorded the absence of new or increased ancillary features favoring viability on posttreatment 3–6 months MRI (*n* = 6), no treated lesion in the LR-TR nonprogressing category was upgraded to the viable category. Regarding imaging features, 47.7% (31/65) of treated lesions had masslike enhancement that was decreased in size over time after SIRT, 1.5% (1/65) had complete lesion disappearance, 13.8% (9/65) had no lesional enhancement, 32.3% (21/65) had smooth perilesional enhancement, and 92.3% (60/65) had parenchymal perfusional changes. Median sizes of whole and viable lesions were 4.6 cm (IQR, 3.5–5.5 cm) and 0.0 cm (IQR, 0.0–1.2 cm), respectively.

### Temporal evolution of the early LR-TRA on multiphase CT/MRI

#### Final LR-TR categories after ≥ 12 months of follow-up

**Fig. 2:**
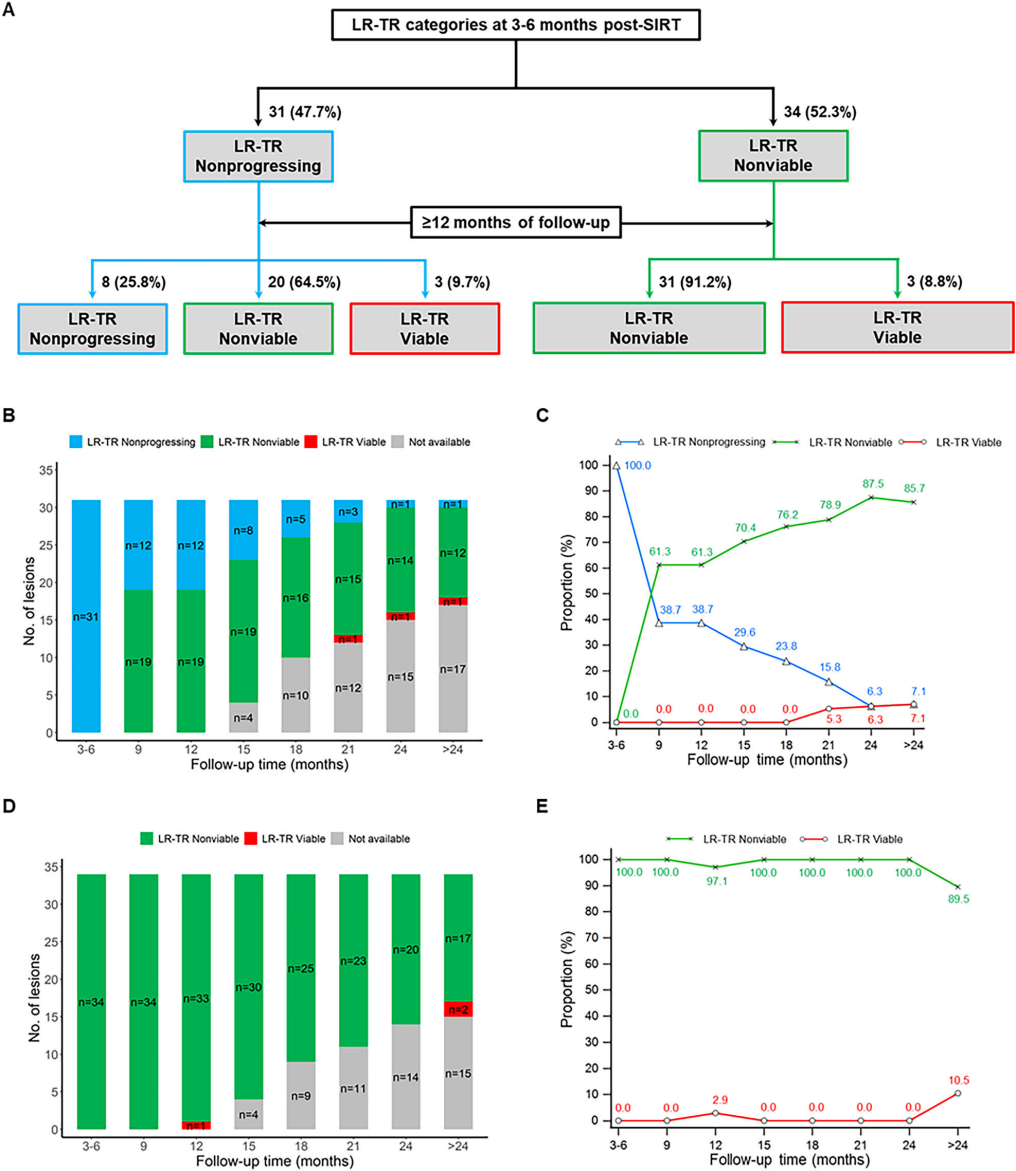
**A** LR-TR category according to the LI-RADS CT/MRI Radiation TRA version 2024 at 3–6 months and ≥ 12 months follow-up multiphase CT/MRI post-SIRT. **B**–**E** Temporal evolution of the early (3–6 months) LR-TR nonprogressing and nonviable categories on multiphase CT/MRI. Histograms show the number of treated lesions with different response statuses at various time points after SIRT, and line graphs show the corresponding proportions of treated lesions for (**B**, **C**) the early LR-TR nonprogressing category (*n* = 31) and (**D**, **E)** the early LR-TR nonviable category (*n* = 34), respectively. CT, computed tomography; LI-RADS/LR, liver imaging reporting and data system; MRI, magnetic resonance imaging; SIRT, selective internal radiation therapy; TR, treatment response; TRA, treatment response algorithm

For the 31 treated lesions assigned to the early nonprogressing category, 64.5% (20/31) regressed to the nonviable category, 25.8% (8/31) remained nonprogressing, and 9.7% (3/31) progressed to the viable category at ≥ 12 months. For the 34 treated lesions assigned to the early nonviable category, 91.2% (31/34) remained nonviable at ≥12 months, and 8.8% (3/34) evolved into the viable category (Fig. 2A).

#### Temporal evolution of the early LR-TR nonprogressing category (*n* = 31)

**Fig. 3:**
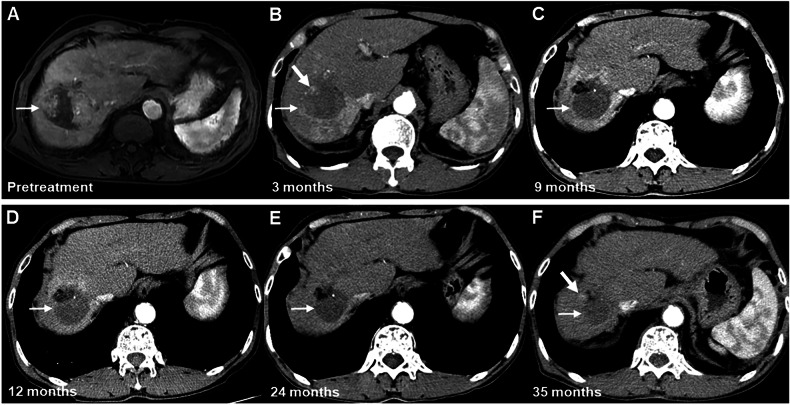
Images of a 76-year-old man with HCC treated by SIRT. MRI or CT obtained during the arterial phase. **A** Pretreatment contrast-enhanced MRI scan shows a 6.2 cm clinically diagnosed HCC (arrow) at segments 7 and 8 of the liver. **B** On the posttreatment contrast-enhanced CT scan performed at 3 months after SIRT, masslike enhancement (thick arrow) was seen and decreased in size in the treated lesion (thin arrow), and this lesion was assigned to the LR-TR nonprogressing category. **C**–**E** On 9–24-month follow-up CT scans, no mass-like enhancement was seen in the treated lesion (arrows) or along its margin; therefore, this lesion was assigned to the LR-TR nonviable category during this period. **F** On a 35-month follow-up CT scan, however, there was a 1.1 cm newly appeared masslike enhancement (thick arrow) along the margin of the treated lesion (thin arrow); thus, this lesion evolved into the LR-TR viable category. CT, computed tomography; HCC, hepatocellular carcinoma; LR, liver imaging reporting and data system; MRI, magnetic resonance imaging; SIRT, selective internal radiation therapy; TR, treatment response

**Fig. 4:**
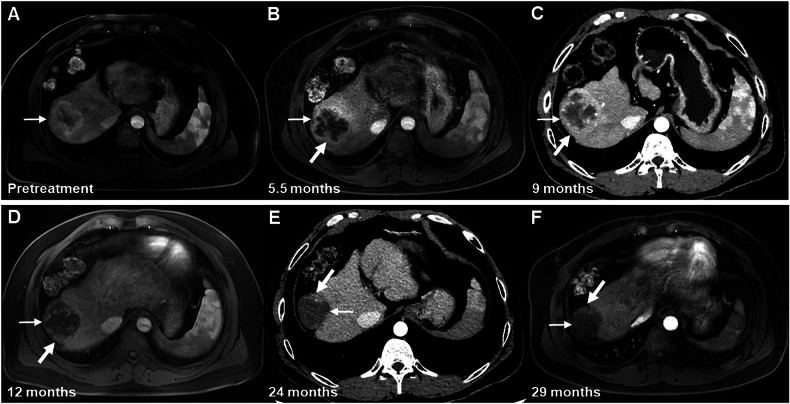
Images of a 34-year-old man with HCC treated by SIRT. MRI or CT obtained during the arterial phase. **A** Pretreatment contrast-enhanced MRI scan shows a 5.7 cm clinically diagnosed HCC (arrow) at segment 8 of the liver. **B** On the posttreatment contrast-enhanced MRI scan performed at 5.5 months after SIRT, masslike enhancement (thick arrow) was seen and decreased in size in the treated lesion (thin arrow), and this lesion was assigned to the LR-TR nonprogressing category. **C**–**F** On 9–29-month follow-up CT or MRI scans, masslike enhancement (thick arrows) was seen and further decreased in size or stable in the treated lesion (thin arrows); thus, this lesion remained in the LR-TR nonprogressing category during this period. CT, computed tomography; HCC, hepatocellular carcinoma; LR, liver imaging reporting and data system; MRI, magnetic resonance imaging; SIRT, selective internal radiation therapy; TR, treatment response

During the follow-up period, the proportion of the nonprogressing category decreased to 38.7% (12/31) at 12 months, 23.8% (5/21) at 18 months, 6.3% (1/16) at 24 months, and 7.1% (1/14) at > 24 months. By contrast, the proportion of the nonviable category increased to 61.3% (19/31) at 12 months, 76.2% (16/21) at 18 months, 87.5% (14/16) at 24 months, and 85.7% (12/14) at > 24 months. The proportion of the viable category slightly increased to 5.3% (1/19) at 21 months, 6.3% (1/16) at 24 months, and 7.1% (1/14) at > 24 months (Fig. 2B, C). Two representative cases demonstrating the temporal evolution of the early LR-TR non-progressing category are presented in Figs. 3 and 4.

#### Temporal evolution of the early LR-TR nonviable category (*n* = 34)

**Fig. 5:**
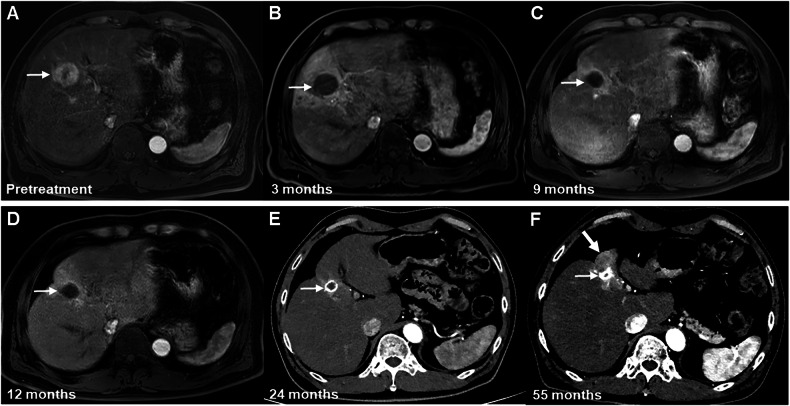
Images of a 61-year-old man with HCC treated by SIRT. MRI or CT obtained during the arterial phase. **A** Pretreatment contrast-enhanced MRI scan shows a 3.7 cm clinically diagnosed HCC (arrow) at segment 4 of the liver. **B** On the posttreatment contrast-enhanced MRI scan performed at 3 months after SIRT, no masslike enhancement was seen in the treated lesion (arrow) or along its margin; therefore, this lesion was assigned to the LR-TR nonviable category. **C**–**E** On 9–24-month follow-up CT or MRI scans, no masslike enhancement was seen in the treated lesion (arrows) or along its margin; instead, smooth perilesional enhancement was observed on (**C**) 9- and (**D**)12-month follow-up MRI scans, and smooth uniform calcification along the lesion (arrow) was observed on (**E**) 24-month follow-up CT scan. Hence, this lesion remained in the LR-TR nonviable category for a long period. **F** On 55-month follow-up CT scan, however, there was a 2.6 cm newly appeared masslike enhancement (thick arrow) along the margin of the treated lesion (thin arrow); thus, this lesion evolved into the LR-TR viable category. CT, computed tomography; HCC, hepatocellular carcinoma; LR, liver imaging reporting and data system; MRI, magnetic resonance imaging; SIRT, selective internal radiation therapy; TR, treatment response

During the follow-up period, the proportion of the nonviable category decreased to 97.1% (33/34) at 12 months and 89.5% (17/19) at > 24 months. By contrast, the proportion of the viable category slightly increased to 2.9% (1/34) at 12 months and 10.5% (2/19) at > 24 months (Fig. 2D, E). A representative case demonstrating the temporal evolution of the early LR-TR nonviable category is presented in Fig. 5.

### Sensitivity analyses for patients with ≥ 24 months of follow-up

Among patients who were followed up for ≥ 24 months (*n* = 36), the evolution of the early LR-TRA on multiphase CT/MRI post-SIRT is shown in Fig. S1.

#### Final LR-TR categories after ≥ 24 months of follow-up

For the 16 treated lesions assigned to the early nonprogressing category, 81.3% (13/16) regressed to the nonviable category, 6.3% (1/16) remained nonprogressing, and 12.5% (2/16) progressed to the viable category at ≥ 24 months. For the 20 treated lesions assigned to the early nonviable category, 90.0% (18/20) remained nonviable at ≥ 24 months, and 10.0% (2/20) evolved into the viable category (Fig. S1A).

#### Temporal evolution of the early LR-TR nonprogressing category (*n* = 16)

During the follow-up period, the proportion of the nonprogressing category decreased to 25.0% (4/16) at 12 months, 18.8% (3/16) at 18 months, 6.3% (1/16) at 24 months, and 7.1% (1/14) at > 24 months. By contrast, the proportion of the nonviable category increased to 75.0% (12/16) at 12 months, 81.3% (13/16) at 18 months, 87.5% (14/16) at 24 months, and 85.7% (12/14) at > 24 months. The proportion of the viable category slightly increased to 6.3% (1/16) at 24 months and 7.1% (1/14) at > 24 months (Fig. S1B, C).

#### Temporal evolution of the early LR-TR nonviable category (*n* = 20)

During the follow-up period, the proportion of the nonviable category decreased to 89.5% (17/19) at > 24 months, whereas the proportion of the viable category increased to 10.5% (2/19) (Fig. S1D, E).

### Subgroup analyses per the BCLC stage (A vs B/C)

Results of subgroup analyses per the BCLC stage (A vs B/C) are shown in the Supplementary Material 2 and Figs. S2 and S3.

### Retreatment for lesions progressing to the LR-TR viable category

For the six treated lesions that progressed to the LR-TR viable category (three treated lesions assigned as nonprogressing and three treated lesions as nonviable at 3–6 months), 50.0% (3/6) were retreated with TACE, 33.3% (2/6) with SIRT, and 16.7% (1/6) with radiofrequency ablation.

### Interobserver agreement on 3–6 months follow-up multiphase CT/MRI

Interobserver agreement among 3 readers for the evaluation of 3–6 months LR-TR category and individual imaging features on CT/MRI is presented in Table 3.

**Table 3:** Interobserver agreement among three radiologists for the evaluation of the 3–6 months LR-TR category and individual imaging features on CT/MRI

Characteristic	CT group (*n* = 47)		MRI group (*n* = 18)		All patients (*n* = 65)	
	κ value	Proportion of agreement	κ value	Proportion of agreement	κ value	Proportion of agreement
LR-TR category	0.46 (0.30, 0.63)	59.6% (44.3%, 73.6%) [28/47]	0.85 (0.50, 1.00)	88.9% (65.3%, 98.6%) [16/18]	0.57 (0.43, 0.71)	67.7% (54.9%, 78.8%) [44/65]
Masslike enhancement	0.46 (0.30, 0.63)	59.6% (44.3%, 73.6%) [28/47]	0.85 (0.50, 1.00)	88.9% (65.3%, 98.6%) [16/18]	0.57 (0.43, 0.71)	67.7% (54.9%, 78.8%) [44/65]
Complete lesion disappearance	0.97 (0.94, 1.00)	97.9% (88.7%, 99.9%) [46/47]	…	100.0% (76.8%, 100.0%) [18/18]	0.98 (0.95, 1.00)	98.5% (91.7%, 100.0%) [64/65]
No lesional enhancement	0.28 (0.12, 0.45)	63.8% (48.5%, 77.3%) [30/47]	0.34 (0.07, 0.61)	83.3% (58.6%, 96.4%) [15/18]	0.30 (0.16, 0.44)	69.2% (56.6%, 80.1%) [45/65]
Smooth perilesional enhancement	0.59 (0.43, 0.76)	74.5% (59.7%, 86.1%) [35/47]	0.62 (0.35, 0.88)	72.2% (46.5%, 90.3%) [13/18]	0.61 (0.46, 0.75)	73.8% (61.5%, 84.0%) [48/65]
Parenchymal perfusional changes	0.77 (0.68, 0.92)	83.0% (69.2%, 92.4%) [39/47]	0.63 (0.41, 0.87)	72.2% (46.5%, 90.3%) [13/18]	0.73 (0.61, 0.86)	80.0% (68.2%, 88.9%) [52/65]
Whole lesion size (cm)^†^	0.87 (0.80, 0.92)	…	0.98 (0.95, 0.99)	…	0.92 (0.88, 0.95)	…
Viable lesion size (cm)^†^	0.57 (0.41, 0.71)	…	0.91 (0.81, 0.96)	…	0.75 (0.65, 0.83)	…

Data in parentheses are 95% confidence intervals

*CT* computed tomography, *LR* liver imaging reporting and data system, *MRI* magnetic resonance imaging, *TR* treatment response

^†^ Data are intraclass correlation coefficients

Specifically, interobserver agreement for the LR-TR category assignment was moderate (kappa = 0.46; 95% confidence interval [CI]: 0.30, 0.63) for CT (*n* = 47), but almost perfect (kappa = 0.85; 95% CI: 0.50, 1.00) for MRI (*n* = 18). Fair interobserver agreement was observed for no lesional enhancement for both CT and MRI (CT: kappa = 0.28, 95% CI: 0.12, 0.45; MRI: kappa = 0.34, 95% CI: 0.07, 0.61). Interobserver agreement for the remaining LR-TR features was moderate to almost perfect (kappa = 0.46–0.97) for CT, and substantial to almost perfect (kappa = 0.62–0.85) for MRI. The proportions of agreement for the LR-TR category and individual imaging features ranged from 59.6% to 97.9% based on CT and from 72.2% to 100.0% based on MRI. Almost perfect interobserver agreement was observed for the whole lesion size based on both CT and MRI (CT: ICC = 0.87, 95% CI: 0.80, 0.92; MRI: ICC = 0.98, 95% CI: 0.95, 0.99). For viable lesion size, interobserver agreement was moderate (ICC = 0.57, 95% CI: 0.41, 0.71) for CT, but almost perfect (ICC = 0.91, 95% CI: 0.81, 0.96) for MRI.

**Fig. 6:**
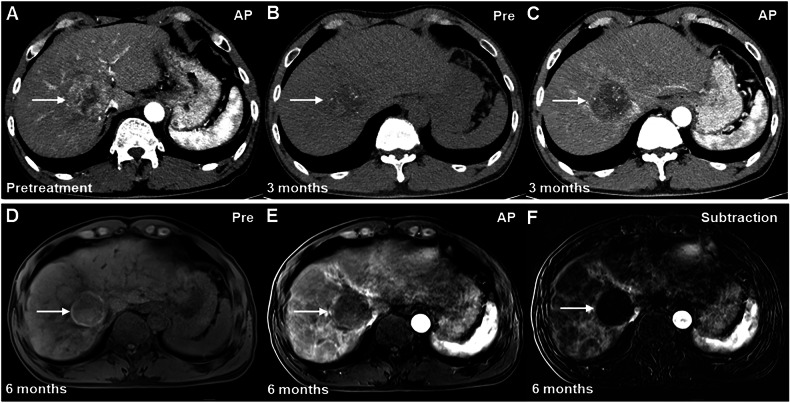
Images of a 59-year-old man with HCC treated by SIRT. **A** Pretreatment contrast-enhanced MRI scan shows a 5.0 cm clinically diagnosed HCC (arrow) at segment 8 of the liver. **B**, **C** On the posttreatment contrast-enhanced CT scan performed at 3 months after SIRT, scattered hyperdense nodules were seen in the treated lesion (arrow) on (**B**) precontrast image. Based on the consensus interpretations, mass-like enhancement was seen and decreased in size in the treated lesion, and this lesion was assigned to the LR-TR nonprogressing category. **D**–**F** On the 6-month follow-up MRI scan, a hyperintense lesion (arrow) was seen on (**D**) T1 precontrast image. The absence of masslike enhancement in the treated lesion (arrow) or along its margin was confirmed by (**F**) arterial phase subtraction imaging; therefore, this lesion was assigned to the LR-TR nonviable category during this period. The treated lesion demonstrated persistent LR-TR nonviable category without evidence of tumor progression on subsequent imaging surveillance from 9 to 46 months. CT, computed tomography; HCC, hepatocellular carcinoma; LR, liver imaging reporting and data system; MRI, magnetic resonance imaging; SIRT, selective internal radiation therapy; TR, treatment response

A representative case utilizing MRI with subtraction imaging and CT for TR assessment is illustrated in Fig. 6.

## Discussion

Assessing tumor response after radiation-based LRTs is challenging due to distinct mechanisms compared to nonradiation LRTs and unique enhancement patterns of treated tumors and surrounding liver parenchyma [22]. The updated 2024 version of the CT/MRI TRA algorithm newly introduced the LR-TR nonprogressing category tailored for radiation-based LRTs, highlighting the importance of serial evaluations to improve the identification of viable diseases and refine treatment decisions [22]. In our evaluation of the LI-RADS CT/MRI Radiation TRA version 2024, the vast majority of treated lesions were either non-viable (52.3%; 34/65) or non-progressing (47.7%; 31/65) at 3–6 months post SIRT, but with gradual increase in proportion of non-viable lesions over time (78.5% [51/65] at ≥ 12 months), and with a consistently low-rate of progression to viable tumors (9.2% [6/65] at ≥ 12 months). Except for fair agreement observed for no lesional enhancement (CT: Kappa = 0.28; MRI: Kappa = 0.34), interobserver agreement was moderate to almost perfect based on CT (Kappa = 0.46–0.97; ICC = 0.57 and 0.87), and substantial to almost perfect based on MRI (Kappa = 0.62–0.85; ICC = 0.91 and 0.98) at 3–6 months.

SIRT induced a delayed imaging response in a significant proportion of our patients, with 64.5% of early nonprogressing lesions evolving into nonviable lesions after ≥ 12 months. This aligns with a prior study on stereotactic body radiation therapy (SBRT), which showed that longer intervals to transplant after SBRT led to greater loss of arterial phase hyperenhancement and an increasing degree of pathologic necrosis [23]. Such a time-dependent response can be attributed to radiation-induced cell death following cellular senescence caused by damage to tumor DNA. In this scenario, the treated tumor remains viable and metabolically active but is unable to replicate. Hence, cell death occurs slowly, resulting in persistent mass-like enhancement within the radiation-treated tumor [24]. Moreover, as a microembolic procedure, SIRT minimally induces flow-related macroscopic embolization and ischemia, thereby preserving tumor perfusion and delaying necrosis [5, 25]. These findings highlight the need for cautious interpretation of early LR-TR nonprogressing lesions to avoid overdiagnosing residual viable tumors and unnecessary retreatments. Close radiologic monitoring, along with reference to serum tumor markers, may be beneficial in this context. Notably, a significant proportion (61.3%) of nonprogressing lesions converted to nonviable by 9 months, suggesting that this duration may serve as a minimum follow-up period for assessing treatment efficacy.

On the other hand, a large proportion of our patients who achieved early tumor nonviability hold a sustained response after SIRT, with 91.2% remaining free of viability at ≥ 12 months. This durable antitumor effect probably stems from the sustained radiation dose delivered by embedded microspheres. Stocker D et al reported a 98–100% positive predictive value of early (around 6 weeks) TR per the LR-TRA version 2018 on MRI for predicting 12-month complete response per the modified Response Evaluation Criteria in Solid Tumors (mRECIST) in HCC treated with radiation segmentectomy [26]. However, the higher radiation dose used in the prior study might have led to more rapid and extensive tumor necrosis compared to the current study. Radiologically nonviable lesions strongly correlate with pathological nonviability in HCC treated with radiation therapy [23, 27], and the latter has been associated with improved survival post-SIRT [28, 29]. Noteworthily, both early non-progressing lesions and non-viable lesions exhibited comparable long-term outcomes in terms of tumor progression, indicating that the differentiation between these categories may have limited clinical relevance.

Interobserver reproducibility remains a challenge in imaging-based response assessment [30]. For the LI-RADS Radiation TRA version 2024, we observed moderate (kappa = 0.57) interobserver agreement for the 3–6 months LR-TR category assignment in all patients, which echoed the results of prior SIRT studies on LI-RADS TRA version 2018 with CT or MRI (kappa = 0.40–81) [26, 27, 31–33]. Interestingly, we further observed moderate (kappa = 0.46) agreement for CT but almost perfect (kappa = 0.85) agreement for MRI regarding the LR-TR category assignment at 3–6 months. In addition, MRI demonstrated superior agreement over CT in terms of masslike enhancement (kappa = 0.85 vs 0.46), no lesional enhancement (kappa = 0.34 vs 0.28), smooth perilesional enhancement (kappa = 0.62 vs 0.59), whole lesion size (ICC = 0.98 vs 0.87), and viable lesion size (ICC = 0.91 vs 0.57). These differences can be attributed to a variety of factors. First, the superior soft tissue contrast resolution of MRI enhances the detection of residual tumors and delineation of tumor margins [34, 35]. Second, non-dynamic imaging sequences, like T2-weighted imaging, diffusion-weighted imaging, and hepatobiliary phase imaging, further aid in distinguishing viable tumors from necrosis and peritumoral parenchymal inflammation. Third, subtraction imaging mitigates the confounding effects of intralesional hemorrhage and calcification and enhances reader confidence for assessment of tumor necrosis following SIRT [29], particularly for less experienced radiologists. Consequently, MRI might be superior to CT in the early detection of LR-TR nonviable lesions, potentially reducing overdiagnosis of residual viable tumors. These advantages make MRI with the subtraction technique preferable over CT for more accurate and consistent long-term monitoring, especially for nonprogressing lesions, to prevent premature retreatment decisions.

This study had several limitations. First, as a single-center retrospective study with a small sample size (*n* = 65), our findings may be subject to selection bias. The particularly limited number of MRI examinations (*n* = 18) calls for cautious interpretation of our preliminary findings. Future larger-scale, multicenter studies incorporating more comprehensive image analyses within an extended follow-up period are warranted, with particular emphasis on evaluating and potentially improving interobserver agreement of LI-RADS radiation TRA across different imaging modalities. Second, using follow-up imaging interpretation as the reference standard for evaluating the evolution of the LR-TRA may have introduced bias, given the inherent inability of imaging to depict microscopic or small foci of residual tumor. Third, only the largest tumor was analyzed in patients with multiple HCCs (*n* = 18, 27.7%), potentially leading to a selection bias, albeit reducing the influence from the clustering data. Fourth, the inclusion of different imaging modalities introduced heterogeneity, although this reflects the real clinical practice. Fifth, the implementation of a minimum 12-month follow-up period for patient eligibility inherently introduces immortal time bias, which may potentially overestimate treatment outcomes. Consequently, our findings should be interpreted with caution, they may not be applicable to patients who underwent additional treatment for residual tumors or progressive diseases within 12 months following SIRT. At last, while the current study elaborated the temporal evolution of the LI-RADS radiation TRA in SIRT-treated HCC lesions, it may not profile the whole spectrum of the patient-level TR. Hence, the interpretation of our findings necessitates careful consideration within the real-world clinical context. Future studies that incorporate both lesion-level (i.e., LI-RADS radiation TRA) and patient-level (e.g., mRECIST) assessment criteria are encouraged to provide a more comprehensive understanding of treatment outcomes.

In conclusion, SIRT induced a delayed and sustained response in the majority of our patients, emphasizing the necessity of dynamic evaluation of long-term changes in treated HCCs. MRI with subtraction imaging may be preferred over CT for more accurate and consistent long-term monitoring, particularly in patients with nonprogressing lesions, which may help avoid premature retreatment decisions following SIRT.

## Supplementary information

ELECTRONIC SUPPLEMENTARY MATERIAL

## Data Availability

Data generated or analyzed during the study are available from the corresponding author by request.

## Abbreviations

AFP: α-fetoprotein
BCLC: Barcelona clinic liver cancer
CI: Confidence interval
HCC: Hepatocellular carcinoma
ICC: Intraclass correlation coefficient
IQR: Interquartile range
LI-RADS/LR: Liver imaging reporting and data system
LRT: Locoregional therapy
mRECIST: modified Response Evaluation Criteria in Solid Tumors
PIVKA-II: Protein induced by vitamin K absence-II
SBRT: Stereotactic body radiation therapy
SIRT: Selective internal radiation therapy
TACE: Transarterial chemoembolization
Tc-99m MAA: Technetium 99m macroaggregated albumin mapping
TR: Treatment response
TRA: Treatment response assessment
Y90: Yttrium-90
