# Supplementary material for: Temporal evolution of the LI-RADS radiation treatment response assessment on multiphase CT/MRI in patients undergoing selective internal radiation therapy for hepatocellular carcinoma
Source: Eur Radiol. 2025 May 17;35(11):7349–62. doi: 10.1007/s00330-025-11659-1 (PMC12559135; doi:10.1007/s00330-025-11659-1)
Supplement: Supplementary file 1 — ELECTRONIC SUPPLEMENTARY MATERIAL [file 330_2025_11659_MOESM1_ESM.docx]

**Supplementary Materials**

**Supplementary Material 1.** Image Technique (Pages 1-2)

**Supplementary Material 2.** Subgroup Analyses per the BCLC Stage (A vs B/C) (Pages 3-4)

**Table S1** MRI Sequences and Parameters (Page 5)

**Fig. S1** Sensitivity Analyses for Patients with ≥24 Months of Follow-up (n = 36) (Page 6)

**Fig. S2** Temporal Evolution of the Early LR-TRA on Multiphase CT/MRI for Patients with BCLC Stage A HCC (Page 7)

**Fig. S3** Temporal Evolution of the Early LR-TRA on Multiphase CT/MRI for Patients with BCLC Stage B/C HCC (Page 8)

**Supplementary Material 1.** Image Technique

***CT Image Acquisition***

CT examinations were conducted using various multidetector row CT scanners, including 16-channel scanners (Sensation 16, Siemens Healthineers, Forchheim, Germany), 64-channel scanners (IQon and Brilliance 64, Philips Healthcare, Best, the Netherlands; Somatom Definition, Siemens Healthineers, Forchheim, Germany; and Discovery CT750 HD, GE Healthcare, Milwaukee, WI), 96-channel scanners (Somatom Force, Siemens Healthineers, Forchheim, Germany), 128-channel scanners (Ingenuity, Philips Healthcare, Best, the Netherlands), and a 256-channel scanner (iCT 256, Philips Healthcare, Best, the Netherlands). The routine liver CT protocol consisted of a precontrast phase, late arterial phase (AP), portal venous phase (PVP), and delayed phase (DP). The AP and PVP images were obtained using the bolus tracking technique with a scan delay of 17–19 seconds and 45–50 seconds, respectively, after a 100 HU trigger threshold was reached in the distal thoracic aorta. Subsequently, DP images were obtained 180 seconds after initiating injection of contrast agent. The acquisition parameters varied by scanners but typically included a slice thickness of 2.5–3.0 mm, a reconstruction interval of 2.0–3.0 mm, a rotation time of 0.5–0.75 seconds, a peak voltage of 80–120 kVp, and a tube current of 150–250 mAs. Patients were scanned craniocaudally following intravenous administration of a nonionic contrast agent, either iobitridol (Xenetix 350, Guerbet, Aulnay-Sous-Bois, France) or iohexol (Bonorex 350, Central Medical Service, Seoul, Korea), at a dose of 1.6 mL/kg and a rate of 3–5 mL/sec followed by a 20–40 mL saline flush using an automatic power injector.

***MRI Acquisition***

MRI examinations were performed on various scanners, including 3.0-T scanners (Skyra, Siemens Healthineers, Erlangen, Germany; Magnetom TrioTim, Siemens Healthineers, Erlangen, Germany; Magnetom Vida, Siemens Healthineers, Erlangen, Germany; Ingenia CX, Philips Healthcare, Best, The Netherlands; Achieva, Philips Healthcare, Best, The Netherlands) and 1.5-T scanners (Signa HDxt, GE Healthcare, Milwaukee, WI; Avanto, Siemens Healthineers, Erlangen, Germany). The routine liver MRI protocol involved a respiratory-triggered T2-weighted fast spin-echo sequence, a half-Fourier acquisition single-shot turbo spin-echo sequence, diffusion-weighted imaging using three b values (50, 400, and 800 s/mm^2^), breath-hold T1-weighted gradient-echo in and out-of-phase sequences, and breath-hold T1-weighted fat-suppressed 3D gradient-echo sequences for precontrast and postcontrast imaging including the arterial phase, portal venous phase (60 s), transitional phase (180 s), and hepatobiliary phase (20 minutes). Using a real-time MR fluoroscopic monitoring system, arterial phase images were acquired 7–8 seconds after contrast agent arrival at the distal thoracic aorta. A standard dose (0.025 mmol/kg) of gadoxetate disodium (Primovist, Bayer Healthcare AG, Leverkusen, Germany) was administered intravenously at a rate of 1.0 mL/sec followed by a 20-mL saline flush using an automatic power injector. MRI acquisition parameters are detailed in **Table S1**.

**Supplementary Material 2.** Subgroup Analyses per the BCLC Stage (A vs B/C)

**Temporal Evolution of the Early LR-TRA on Multiphase CT/MRI for Patients with BCLC Stage A HCC**

Among patients with BCLC stage A HCC (n = 34), the evolution of the early LR-TRA on multiphase CT/MRI post-SIRT is shown in **Fig. S2.**

***Final LR-TR Categories after ≥12 Months of Follow-up***

For the 14 treated lesions assigned into the early nonprogressing category, 78.6% (11/14) regressed to the nonviable category, and 21.4% (3/14) remained nonprogressing at ≥12 months. For the 20 treated lesions assigned into the early nonviable category, 90.0% (18/20) remained nonviable at ≥12 months, and 10.0% (2/20) evolved into the viable category (**Fig. S2A**).

***Temporal Evolution of the*** ***Early*** ***LR-TR Nonprogressing Category (n = 14)***

During the follow-up period, the proportion of the nonprogressing category decreased to 28.6% (4/14) at 12 months, 18.2% (2/11) at 18 months, 0.0% (0/8) at 24 months, and 0.0% (0/7) at >24 months. By contrast, the proportion of the nonviable category increased to 71.4% (10/14) at 12 months, 81.8% (9/11) at 18 months, 100.0% (8/8) at 24 months, and 100.0% (7/7) at >24 months. (**Fig. S2B, C**).

***Temporal Evolution of the Early LR-TR Nonviable Category (n = 20)***

During the follow-up period, the proportion of the nonviable category decreased to 83.3% (10/12) at >24 months. By contrast, the proportion of the viable category slightly increased to 16.7% (2/12) at >24 months (**Fig. S2D, E**).

**Temporal Evolution of the Early LR-TRA on Multiphase CT/MRI for Patients with BCLC Stage B/C HCC**

Among patients with BCLC stage B/C HCC (n = 31), the evolution of the early LR-TRA on multiphase CT/MRI post-SIRT is shown in **Fig. S3.**

***Final LR-TR Categories after ≥12 Months of Follow-up***

For the 17 treated lesions assigned into the early nonprogressing category, 52.9% (9/17) regressed to the nonviable category, 29.4% (5/17) remained nonprogressing, and 17.6% (3/17) progressed to the viable category at ≥12 months. For the 14 treated lesions assigned into the early nonviable category, 92.9% (13/14) remained nonviable at ≥12 months, and 7.1% (1/14) evolved into the viable category (**Fig. S3A**).

***Temporal Evolution of the Early LR-TR Nonprogressing Category (n = 17)***

During the follow-up period, the proportion of the nonprogressing category decreased to 47.1% (8/17) at 12 months, 30.0% (3/10) at 18 months, 12.5% (1/8) at 24 months, and 14.3% (1/7) at >24 months. By contrast, the proportion of the nonviable category increased to 52.9% (9/17) at 12 months, 70.0% (7/10) at 18 months, 75.0% (6/8) at 24 months, and 71.4% (5/7) at >24 months. The proportion of the viable category slightly increased to 11.1% (1/9) at 21 months, 12.5% (1/8) at 24 months, and 14.3% (1/7) at >24 months (**Fig. S3B, C**).

***Temporal Evolution of the Early LR-TR Nonviable Category (n = 14)***

During the follow-up period, the proportion of the nonviable category decreased to 92.9% (13/14) at 12 months and increased to 100.0% (7/7) at >24 months. By contrast, the proportion of the viable category slightly increased to 7.1% (1/14) at 12 months and decreased to 0.0% (0/7) at >24 months (**Fig. S3D, E**).

**Table S1** MRI Sequences and Parameters

| Sequence | T1-weighted IP and OP imaging | Dynamic T1-weighted 3D GRE | T2-weighted 2D FSE | Diffusion-weighted imaging |
| --- | --- | --- | --- | --- |
| **Skyra, Siemens Healthineers, Erlangen, Germany (3.0 T)** | | | | |
| Repetition time (ms) | 4.5 | 3.78 | 2560 | 2000 |
| Echo time (ms) | 1.52 | 1.41 | 100 | 61 |
| Flip angle (°) | 9 | 11 | 111 | 90 |
| Section thickness (mm) | 3 | 3 | 4 | 5 |
| Spacing (mm) | 3 | 3 | 4 | 5.5 |
| Matrix size | 384x307 | 384x384 | 256x256 | 140x112 |
| Field of view (mm^2^) | 380x380 | 380x380 | 380x380 | 400x320 |
| Acquisition time (s) | 46 | 17 | 89 | 189 |
| Fat suppression (Yes/No) | No | Yes | Yes | Yes |
| **Magnetom TrioTim, Siemens Healthineers, Erlangen, Germany (3.0 T)** | | | | |
| Repetition time (ms) | 4 | 2.5 | 6156.6 | 5000 |
| Echo time (ms) | 1.2 | 0.9 | 81 | 67 |
| Flip angle (°) | 9 | 10.9 | 140 | 90 |
| Section thickness (mm) | 3 | 2 | 4 | 5 |
| Spacing (mm) | 3.6 | 2 | 5 | 6 |
| Matrix size | 256x192 | 256x192 | 320x168 | 128x96 |
| Field of view (mm^2^) | 420x315 | 420x315 | 420x315 | 420x315 |
| Acquisition time (s) | 48 | 24 | 75 | 280 |
| Fat suppression (Yes/No) | No | Yes | Yes | Yes |
| **Magnetom Vida, Siemens Healthineers, Erlangen, Germany (3.0 T)** | | | | |
| Repetition time (ms) | 4.3 | 3.3 | 5652.2 | 2200 |
| Echo time (ms) | 1.3 | 1.3 | 94 | 45 |
| Flip angle (°) | 10 | 10 | 106 | 90 |
| Section thickness (mm) | 2.8 | 3 | 4 | 4 |
| Spacing (mm) | 2.8 | 3 | 5 | 5 |
| Matrix size | 288x220 | 352x238 | 400x400 | 140x140 |
| Field of view (mm^2^) | 380x322 | 380x322 | 380x380 | 380x380 |
| Acquisition time (s) | 31 | 8 | 241 | 45 |
| Fat suppression (Yes/No) | No | Yes | Yes | Yes |
| **Ingenia CX, Philips Healthcare, Best, The Netherlands (3.0 T)** | | | | |
| Repetition time (ms) | 4 | 4 | 3560 | 4280 |
| Echo time (ms) | 1.18 | 1 | 80 | 64.1 |
| Flip angle (°) | 10 | 10 | 90 | 90 |
| Section thickness (mm) | 6 | 6 | 4 | 5 |
| Spacing (mm) | 3 | 3 | 4 | 5 |
| Matrix size | 320x290 | 324x272 | 344x212 | 126x126 |
| Field of view (mm^2^) | 380x380 | 380x380 | 380x380 | 380x380 |
| Acquisition time (s) | 60 | 19 | 45 | 352 |
| Fat suppression (Yes/No) | No | Yes | Yes | Yes |
| **Achieva, Philips Healthcare, Best, The Netherlands (3.0 T)** | | | | |
| Repetition time (ms) | 3.4 | 3.1 | 1232.4 | 3108.5 |
| Echo time (ms) | 1.2 | 1.5 | 80 | 57.6 |
| Flip angle (°) | 10 | 10 | 90 | 90 |
| Section thickness (mm) | 5 | 5 | 5 | 5 |
| Spacing (mm) | 5 | 2.5 | 6 | 6 |
| Matrix size | 160x160 | 264x224 | 264x251 | 128x124 |
| Field of view (mm^2^) | 380x380 | 380x380 | 380x380 | 380 x380 |
| Acquisition time (s) | 48 | 16 | 40 | 240 |
| Fat suppression (Yes/No) | No | Yes | Yes | Yes |
| **Signa HDxt, GE Healthcare, Milwaukee, WI (1.5 T)** | | | | |
| Repetition time (ms) | 7.9 | 4.5 | 2050 | 5150 |
| Echo time (ms) | 5.2 | 2.2 | 83.6 | 67.9 |
| Flip angle (°) | 12 | 12 | 90 | 90 |
| Section thickness (mm) | 6 | 6 | 7 | 5 |
| Spacing (mm) | 3 | 3 | 7 | 6 |
| Matrix size | 320x192 | 320x224 | 256x256 | 128x96 |
| Field of view (mm^2^) | 380x380 | 380x380 | 380x380 | 380x380 |
| Acquisition time (s) | 50 | 21 | 49 | 342 |
| Fat suppression (Yes/No) | No | Yes | Yes | Yes |
| **Avanto, Siemens Healthineers, Erlangen, Germany (1.5 T)** | | | | |
| Repetition time (ms) | 7.6 | 3.6 | 4537.1 | 5700 |
| Echo time (ms) | 2.4 | 1.6 | 269 | 81 |
| Flip angle (°) | 10 | 12 | 120 | 90 |
| Section thickness (mm) | 3 | 3 | 3 | 5 |
| Spacing (mm) | 3 | 3 | 3 | 6.5 |
| Matrix size | 320x114 | 320x174 | 384x290 | 190x172 |
| Field of view (mm^2^) | 380x285 | 380x344 | 384x290 | 380x344 |
| Acquisition time (s) | 30 | 20 | 330 | 120 |
| Fat suppression (Yes/No) | No | Yes | Yes | Yes |
| Note.—*FSE* fast spin-echo, *GRE* gradient recall echo, *IP* in-phase, *MRI* magnetic resonance imaging, *OP* opposed-phase, *2D* two-dimensional, *3D* three-dimensional | | | | |


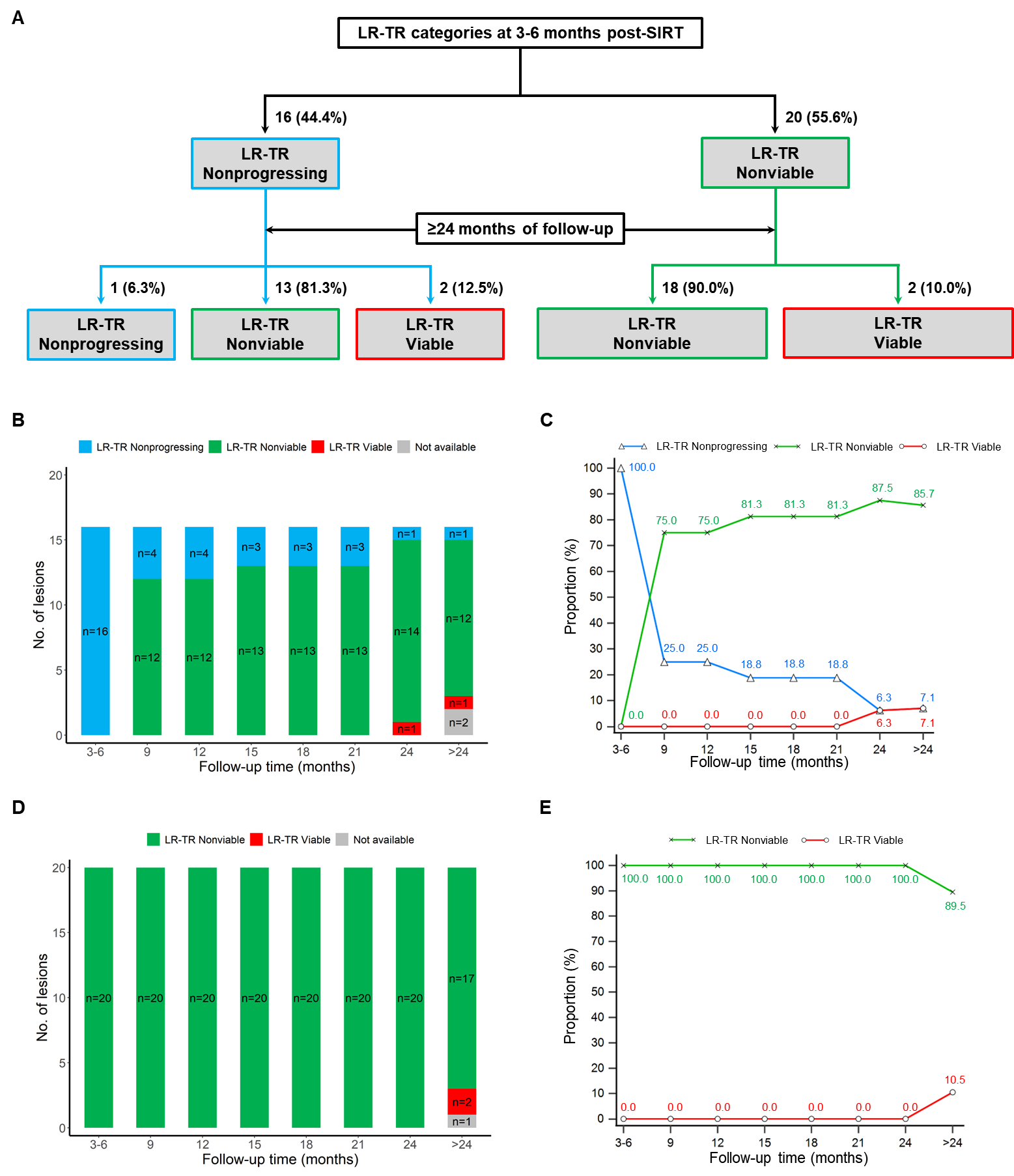


**Fig. S1** Sensitivity analyses for patients with ≥24 months of follow-up (n = 36). **A** LR-TR categories according to the LI-RADS CT/MRI Radiation TRA version 2024 at 3-6 months and ≥24 months follow-up multiphase CT/MRI post-SIRT. **B-E** Temporal evolution of the early (3-6 months) LR-TR nonprogressing and nonviable categories on multiphase CT/MRI. Histograms show the number of treated lesions with different response statuses at various time points after SIRT and line graphs show the corresponding proportions of treated lesions for (**B, C)** the early LR-TR nonprogressing category (n = 16) and **(D, E)** the early LR-TR nonviable category (n = 20), respectively. CT, computed tomography; LI-RADS/LR, Liver Imaging Reporting and Data System; MRI, magnetic resonance imaging; SIRT, selective internal radiation therapy; TR, treatment response; TRA, treatment response algorithm


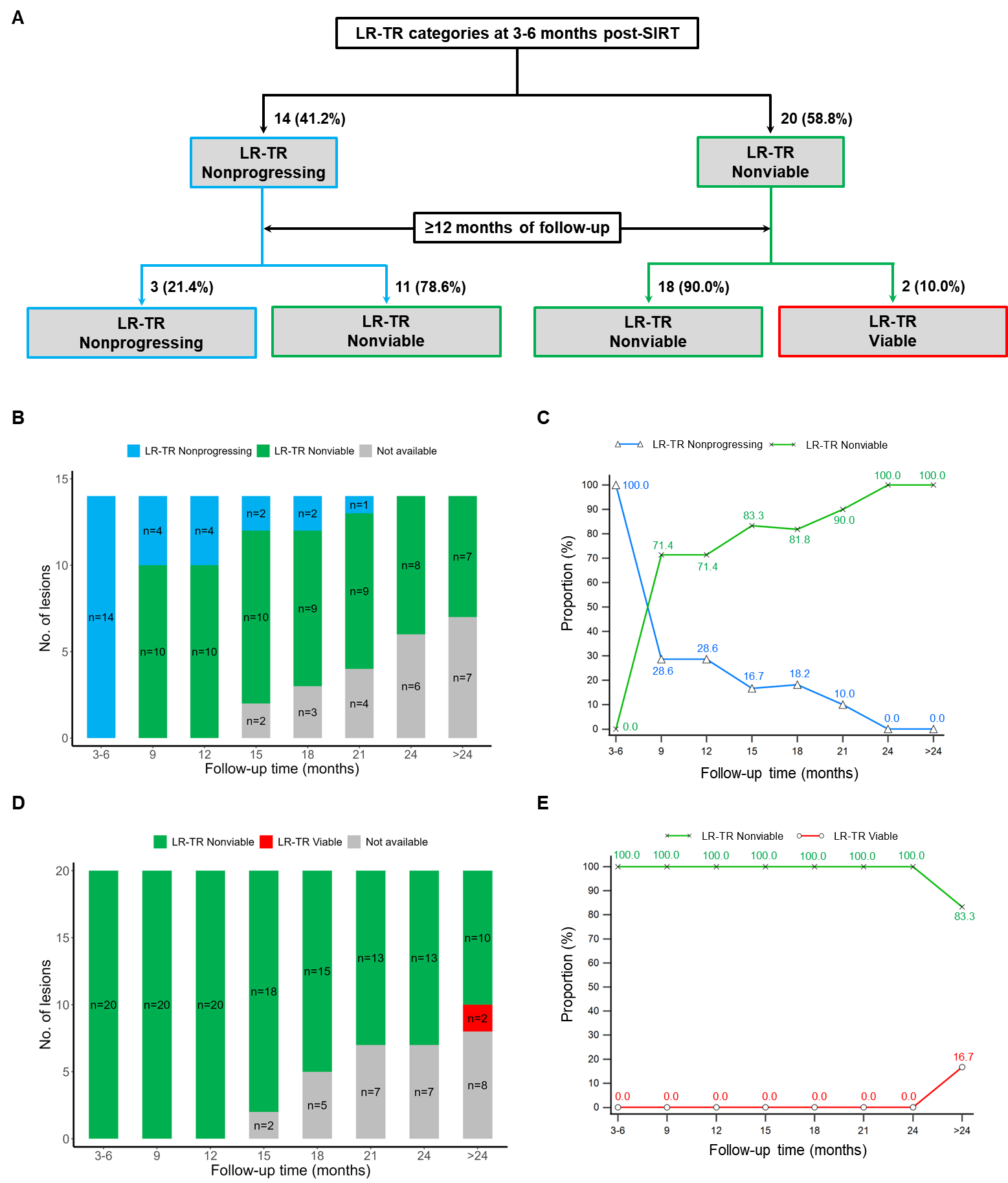


**Fig. S2** Subgroup analyses for patients with BCLC stage A HCC (n = 34). **A** LR-TR categories according to the LI-RADS CT/MRI Radiation TRA version 2024 at 3-6 months and ≥12 months follow-up multiphase CT/MRI post-SIRT. **B-E** Temporal evolution of the early (3-6 months) LR-TR nonprogressing and nonviable categories on multiphase CT/MRI. Histograms show the number of treated lesions with different response statuses at various time points after SIRT and line graphs show the corresponding proportions of treated lesions for (**B, C)** the early LR-TR nonprogressing category (n = 14) and **(D, E)** the early LR-TR nonviable category (n = 20), respectively. BCLC, Barcelona Clinic Liver Cancer; CT, computed tomography; HCC, hepatocellular carcinoma; LI-RADS/LR, Liver Imaging Reporting and Data System; MRI, magnetic resonance imaging; SIRT, selective internal radiation therapy; TR, treatment response; TRA, treatment response algorithm


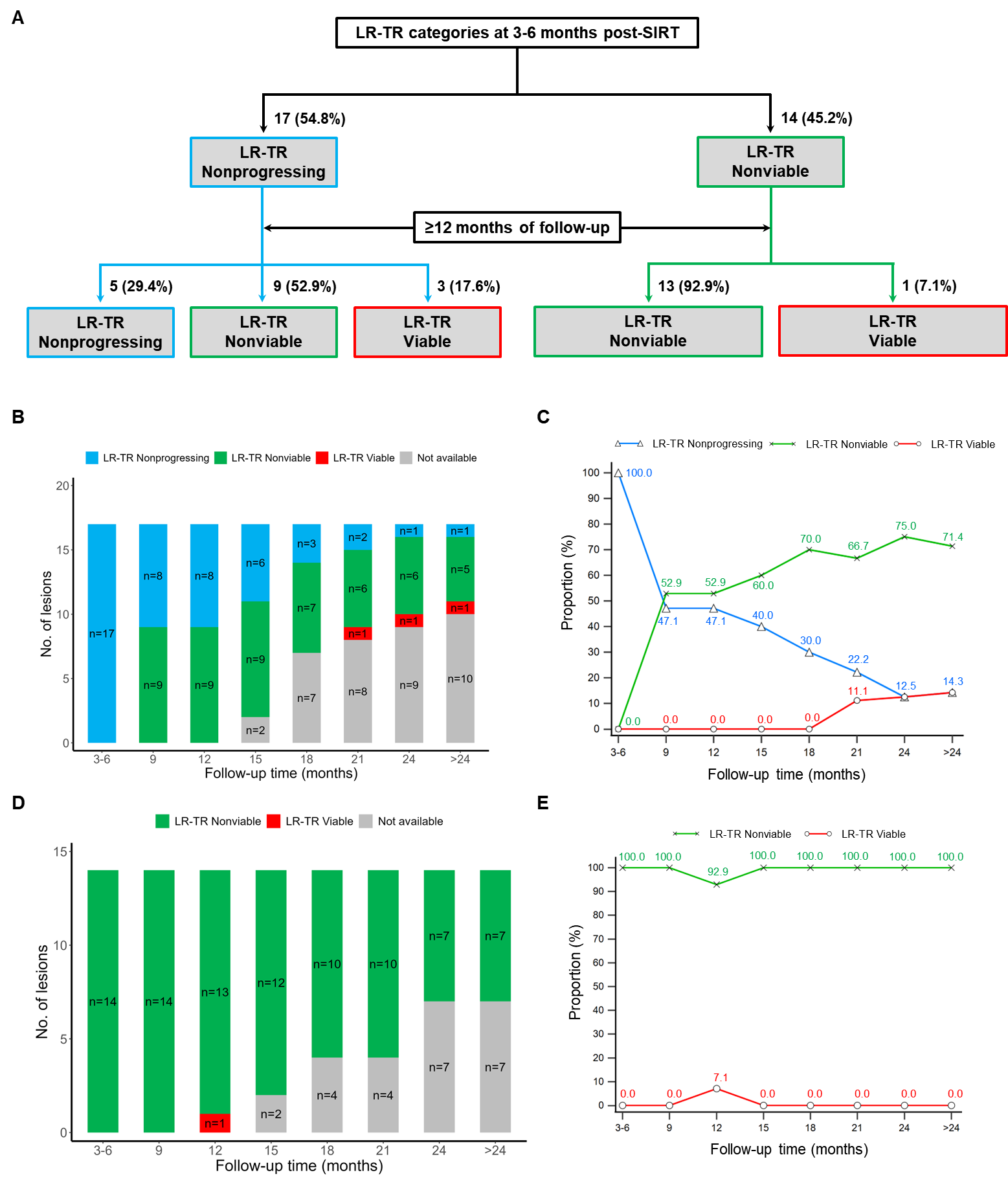


**Fig. S3** Subgroup analyses for patients with BCLC stage B/C HCC (n = 31). **A** LR-TR categories according to the LI-RADS CT/MRI Radiation TRA version 2024 at 3-6 months and ≥12 months follow-up multiphase CT/MRI post-SIRT. **B-E** Temporal evolution of the early (3-6 months) LR-TR nonprogressing and nonviable categories on multiphase CT/MRI. Histograms show the number of treated lesions with different response statuses at various time points after SIRT and line graphs show the corresponding proportions of treated lesions for (**B, C)** the early LR-TR nonprogressing category (n = 17) and **(D, E)** the early LR-TR nonviable category (n = 14), respectively. BCLC, Barcelona Clinic Liver Cancer; CT, computed tomography; HCC, hepatocellular carcinoma; LI-RADS/LR, Liver Imaging Reporting and Data System; MRI, magnetic resonance imaging; SIRT, selective internal radiation therapy; TR, treatment response; TRA, treatment response algorithm
